# Supplementary material for: Comparative multiomics analysis of cell physiological state after culture in a basket bioreactor
Source: Sci Rep. 2022 Nov 23;12:20161. doi: 10.1038/s41598-022-24687-4 (PMC9686226; doi:10.1038/s41598-022-24687-4)

4:30558043:-@NW_023666068.1:30557564:30557712:-@NW_023666068.1:30556876:30556979:-@NW_0236660

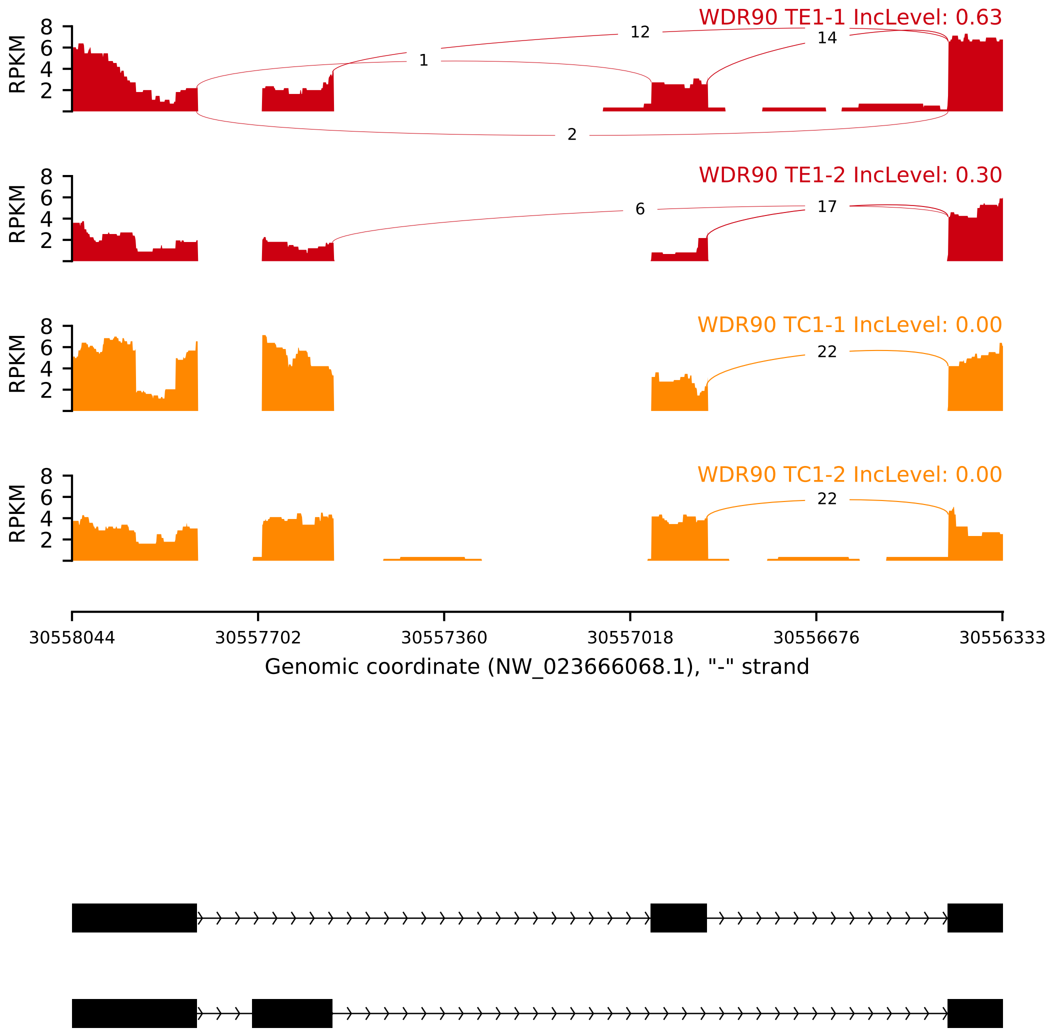

Supplement: Supplementary file 1 — Supplementary Information 1. [file 41598_2022_24687_MOESM1_ESM.zip › raw data/Transcriptomic raw data/7.AS/2.dasplot/TE1vsTC1/MXE/11_WDR90_NW_023666068.1_30557814_30558043_-@NW_023666068.1_30557564_30557712_-@NW_023666068.1_30556876_30556979_-@NW_023666068.1_30556331_30556433_-.pdf]
